# Outcomes of concomitant *Pneumocystis jirovecii* pneumonia and cytomegalovirus co-infection in non-HIV, mechanically ventilated critically ill patients

**DOI:** 10.1080/07853890.2026.2677997

**Published:** 2026-06-05

**Authors:** Wei-Syun Hung, Meng-Jer Hsieh, Shaw-Woei Leu, Meng-Heng Hsieh, Yueh-Fu Fang, Po-Jui Chang, Shu-Min Lin, Horng-Chyuan Lin, Chung-Chi Huang, Ko Cheng, Chun-Yu Lin

**Affiliations:** ^a^ Department of Thoracic Medicine, Chang Gung Memorial Hospital, Linkou, Taoyuan, Taiwan; ^b^ College of Medicine, Chang Gung University, Taiwan; ^c^ Department of Respiratory Therapy, Chang Gung Memorial Hospital, Linkou, Taiwan; ^d^ School of Medicine, National Tsing Hua University, Hsin-Chu, Taiwan; ^e^ Center for Big Data Analytics and Statistics, Chang Gung Memorial Hospital, Taoyuan, Taiwan; ^f^ Department of Thoracic Medicine, Kaohsiung Chang Gung Memorial Hospital, Kaohsiung, Taiwan

**Keywords:** *Pneumocystis jirovecii* pneumonia, cytomegalovirus, critically ill patients

## Abstract

**Purpose:**

*Pneumocystis jirovecii* pneumonia (PJP) is a potentially life-threatening opportunistic infection. Recent studies have demonstrated a poor prognosis and higher mortality rate in non-human immunodeficiency virus (HIV) patients, with associated risk factors including cytomegalovirus (CMV) co-infection. We aimed to investigate the outcomes of concomitant PJP and CMV infection in non-HIV mechanically ventilated critically ill patients.

**Patients and methods:**

We retrospectively enrolled adult patients admitted to an intensive care unit (ICU) and diagnosed with PJP infection from January 2017 to December 2022. Data were retrieved from the Chang Gung Research Database, including clinical manifestations, comorbidities and mortality.

**Results:**

A total of 132 adult patients without HIV infection received mechanical ventilation in the ICU, underwent bronchoalveolar lavage and diagnosed with PJP were enrolled, of whom 26 patients had concomitant CMV infection and 106 did not. The PJP and concomitant CMV infection group had a significantly lower PaO2/FiO2 ratio (73.5 vs. 95.6, *p* = 0.04) and higher procalcitonin level (2.2 ng/ml vs. 0.4 ng/ml, *p* = 0.004). While CMV co-infection was associated with higher ICU mortality in the univariate analysis (33.3% vs. 11.1%, *p* = 0.002), multivariate analysis revealed that systemic CMV co-infection was not an independent predictor of mortality. Instead, the extended model demonstrated that mortality was significantly associated with acute respiratory distress syndrome (ARDS) (HR 4.281, 95% CI:1.178–15.565, *p* = 0.027) and the duration of PJP treatment (HR: 0.892, 95% CI: 0.803–0.990, *p* = 0.006).

**Conclusion:**

Concomitant CMV infection was about one-fifth in the non-HIV critically ill patients with PJP infection but does not independently increase mortality risk. Clinical management should prioritize early, sustained anti-pneumocystis therapy and lung-protective strategies for ARDS.

## Introduction

*Pneumocystis jirovecii* pneumonia (PJP) is a potentially life-threatening opportunistic fungal infection caused by *Pneumocystis jirovecii* (*P. jiroveci* [Pj]). Although human immunodeficiency virus (HIV) infection is a known risk factor for PJP infection [1,2], the incidence of PJP among patients infected with HIV has markedly decreased worldwide because of routine primary PJP prophylaxis [3]. Nevertheless, PJP continues to gain attention due to its significantly increased incidence in patients with haematological and solid organ malignancies, organ transplantation and autoimmune disease who are receiving corticosteroids, biological agents, or immunosuppressants [4–7]. In addition, compared to HIV-infected patients, HIV-negative patients with PJP typically present with rapidly progressive respiratory failure [8–10]. Moreover, the use of invasive ventilation, duration of ventilator use, and overall mortality rate have also significantly risen over time in these patients [5,8,11–13].

Cytomegalovirus (CMV) infection is also an opportunistic infection, mostly occurring in hematopoietic stem cell transplantation (HSCT) and solid organ transplant recipients, for which prophylactic treatment is suggested [14,15]. CMV infection can also occur in immunocompetent critically ill patients, and CMV reactivation has been reported to be an indicator of stronger immunosuppression and illness severity [16]. CMV has been found to reactivate in patients infected with other pathogens, especially *P. jiroveci* [17]. In addition, an animal study suggested that CMV infection inhibits the immune responses generated against *P. jirovecii*, which may contribute to delayed clearance of the organism [18]. Despite these findings and that some studies have reported that PJP patients with CMV co-infection have an increased risk of ICU admission and longer ICU stay [16,19], the comprehensive outcomes of patients with PJP with CMV co-infection are still not well understood, especially with regards to mortality and in critically ill patients with mechanical ventilation [16,20,21].

In this study, we aimed to investigate the manifestations and outcomes of concomitant PJP and CMV infection in non-HIV mechanically ventilated patients.

## Material and methods

### Data source

We used the Chang Gung Research Database (CGRD) to conduct this multicenter retrospective cohort study from 2017 to 2022. The CGRD is the electronic medical records database of the Chang Gung Medical Foundation, which is the largest hospital system in Taiwan, comprising three medical centres (Linkou, Taipei, and Kaohsiung branches) and four regional hospitals (Taoyuan, Keelung, Chiayi, and Yunlin branches).

### Study population

We searched the CGRD and retrieved the data of patients who admitted to ICU because of respiratory failure and having bronchoalveolar lavage (BAL) between 1 January 2017 and 31 December 2022. The inclusion criteria were as follows: 1) aged 18 years or older; 2) without HIV infection; 3) admitted to an intensive care unit (ICU) due to respiratory failure; and 4) received mechanical ventilation. The exclusion criteria were as follows: 1) no BAL performed for PJP testing; 2) absence of a final diagnosis of PJP; and 3) no CMV plasma viral load when admitted to ICU. The diagnosis of PJP was confirmed by the medical record with discharge diagnosis review in combination with BAL PCR testing.

### Sampling methods, microbiologic assessments and treatment

BAL was performed according to consensus guidelines [22–24]. The bronchoscope was placed in a wedged position within the selected bronchopulmonary segment. Sequential aliquots of normal saline totalling at least 100 mL (and no more than 300 mL) were instilled, and at least 30% returned for optimal sampling [22].

Microbiological examinations were performed using real-time PCR to detect *P. jirovecii* in fluid from BAL. We checked mitochondrial large subunit rRNA (mtLSUrRNA) to detect *P. jirovecii* and defined a Ct value ≤ 36 as indicating a high probability of *P. jirovecii* infection [25,26]. CMV was detected in blood samples with a commercial real-time PCR assay (cobas^®^ CMV for cobas^®^ 5800/6800/8800 Systems) when the patients admitted to ICU and had BAL for infection survey. Systemic CMV infection was defined as a plasma viral load ≥ 2000 IU/ml as the diagnostic cutoff value [14]. The threshold values for bacterial quantitative evaluations were 10^5^ cfu/mL for endotracheal aspirate material, and 10^4^ cfu/mL for BAL [27]. Other data retrieved from the CGRD included clinical manifestations, comorbidities, disease severity, complication, treatment outcomes and mortality. Disease severity including Acute Physiology and Chronic Health Evaluation (APACHE) score, PF ratio were evaluated when admitted to ICU. Complications such as acute respiratory distress syndrome (ARDS), septic shock, and the need for renal replacement therapy were recorded during ICU stay.

### Statistical analysis

Categorical and parametric variables are presented as count (percentage) and median [interquartile range], respectively. Two-way categorical variables were compared using the chi–square test. The Mann-Whitney U test was performed to compare continuous variables with abnormal distribution and considering the small number of patients in subgroups. Univariate and multivariate Cox regression analyses were performed to evaluate mortality risk associated with different independent variables, and the results were presented as hazard ratios (HRs) with mortality as the event. Covariates were selected based on clinical relevance, including CMV co-infection, other pulmonary co-infection, follow-up complications, PJP treatment duration, and disease severity. Pearson correlation and variance Inflation was used to test several variables for possible collinearity. Variables that may induce immortal time bias was removed from the multivariate model. To clarify the causal relationship, Cox regression models were constructed: a primary adjusted model including baseline confounders and an extended model additionally including variables that may lie on the causal pathway. Variables such as ARDS, septic shock, and treatment-related factors were considered mediators and were therefore excluded from the primary model. No model adjustment was performed for quasi-complete and complete separation of the data. A *p*-value of 0.05 was considered statistically significant. Data processing and analysis were performed using SAS version 9.4 (SAS Institute, Inc.).

## Results

**Figure 1.**
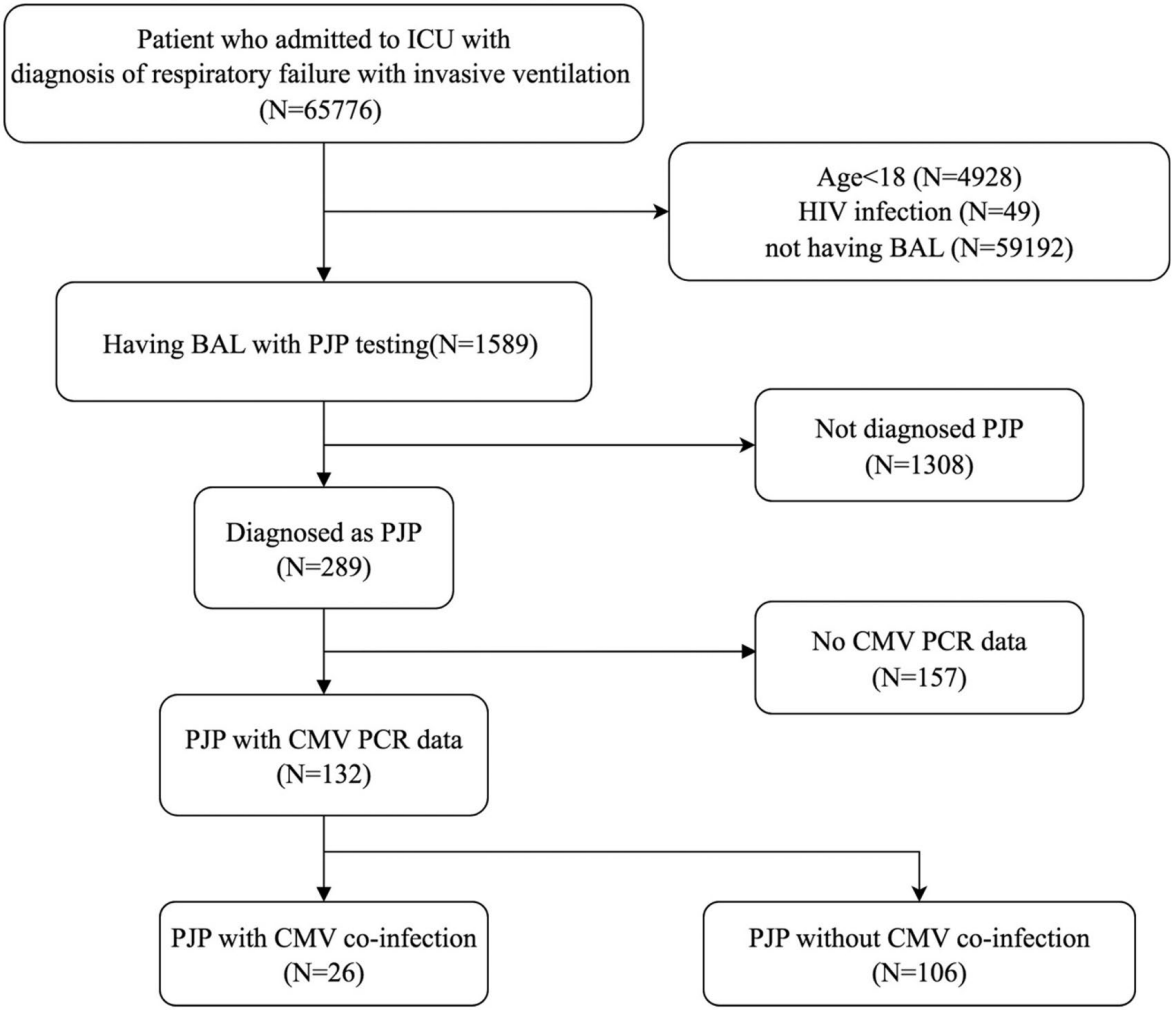
Flow chart of the study population.

A total of 1597 adult patients without HIV infection who received mechanical ventilation in the ICU and underwent BAL with PJP testing were finally enrolled. Of these patients, 289 were diagnosed with PJP during the study period. We excluded 157 patients without CMV PCR data, and of the remaining 132 patients (mean age, 62.5 ± 13.8 years), 26 (19.7%) had concomitant CMV infection and 106 did not experienced concomitant CMV infection (Figure 1).

In addition, of the patients without PJP (*n* = 1308), 416 were tested for serum CMV DNA, and 28 had CMV infection (6.7%). The incidence of CMV infection was significantly different between the patients without PJP but with CMV infection and those with both PJP and CMV infection (6.7% vs. 19.7%, *p* < 0.001). Overall, 45.7% (*n* = 132) of the 289 patients diagnosed with PJP died during the study period.

### Clinical characteristics

The demographic characteristics of the PJP patients with and without CMV co-infection are shown in Table 1. In the overall cohort, the most prevalent underlying immunosuppressive disease was solid malignancy (46.2%) followed by hematologic malignancy (10.6%). There were no significant differences between PJP patients with and without CMV co-infection in sex, age, comorbidities, immunosuppressive status or immunosuppressive drugs. A high proportion of the patients used steroids (75%), and 20% received trimethoprim-sulfamethoxazole (TMP-SMX) for PJP prophylaxis. Three patients were found having COVID-19 infection before ICU admission and two of them have dexamethasone use for COVID-19 treatment.

**Table 1.** Demographic and clinical characteristics of the patients with PJP.

	Total (*n* = 132)	With CMV systemic infection (*n* = 26)	Without CMV systemic infection (*n* = 106)	*p*-value
**Sex (male), n (%)**	88 (66.7)	19 (73.1)	69 (65.1)	0.44
**Age, years (mean ± SD)**	62.5 ± 13.8	63.9 ± 11.1	62.2 ± 14.4	0.56
**Comorbidity, n (%)**				
Heart failure	3 (2.3)	0 (0.0)	3 (2.8)	1
Chronic obstructive pulmonary disease	7 (5.3)	2 (7.7)	5 (4.7)	0.62
Chronic kidney disease	10 (7.6)	1 (3.9)	9 (8.5)	0.69
Diabetes mellitus	1 (0.8)	0 (0.0)	1 (0.9)	1
Liver cirrhosis	4 (3.0)	0 (0.0)	4 (3.8)	0.58
**Immunosuppressive disease, n (%)**				
Connective tissue disease	17 (12.9)	3 (11.5)	14 (13.2)	1
Vasculitis, n (%)	1 (0.8)	0 (0.0)	1 (0.9)	1
Organ transplant, n (%)	6 (4.6)	0 (0.0)	6 (5.7)	0.6
Cancer (Solid organ tumour), n (%)	61 (46.2)	11 (42.3)	50 (47.2)	0.66
Haematological malignancy, n (%)	14 (10.6)	1 (3.9)	13 (12.3)	0.3
**Immunosuppressive therapy, n (%)**				
Systemic Steroid	99 (75)	21 (80.8)	78 (73.6)	0.45
T-cell immunosuppressant	26 (19.7)	6 (23.1)	20 (18.9)	0.63
Tacrolimus	8(6.1)	0(0.0)	8(7.6)	0.35
Sirolimus	1(0.8)	0(0.0)	1(0.9)	1.00
Azathioprine	12(9.1)	4(15.4)	8(7.6)	0.25
Mycophenolate mofetil	7(5.3)	0(0.0)	7(6.6)	0.34
Mycophenolate sodium	5(3.8)	0(0.0)	5(4.7)	0.58
Cyclosporin	9(6.8)	0(0.0)	6(5.7)	0.38
B-cell immunosuppressant	3 (2.3)	0 (0.0)	3 (2.8)	1.00
Rituximab	3(2.3)	0(0.0)	3(2.8)	1.00
Other Immunosuppressant	9 (6.8)	1 (3.9)	8 (7.6)	0.69
**Anti-cancer agent, n (%)**	28 (21.2)	2 (7.7)	26 (24.5)	0.06
**Prophylaxis for PJP**	28 (21.2)	4 (15.4)	24 (22.6)	0.59
**COVID-19 infection with steroid treatment (%)**	2(1.5)	1(3.9)	1(0.9)	0.36

Abbreviations: CMV, cytomegalovirus; PJP, *Pneumocystis jirovecii* pneumonia.

Values are listed as mean ± SD or number (%).

Categorical variables were compared by the chi-square test.

The Mann-Whitney U test was performed to compare continuous variables with abnormal distribution and given the small number of patients in subgroups.

The clinical characteristics on admission to the ICU, including results of blood analysis and microbiological examinations, disease severity and treatment are shown in Table 2. Overall, 94 of the 132 patients (71%) had acute respiratory distress syndrome (ARDS), and there was no significant difference between the PJP patients with or without concomitant CMV infection groups. Although a higher proportion of the PJP with concomitant CMV group experienced septic shock (84.6% vs. 68.9%, *p* = 0.14), there was no significance between the two groups. Regarding disease severity, the PaO_2_/FiO_2_ ratio (PF ratio) was significantly lower in the PJP with concomitant CMV infection group (73.5 vs. 95.6, *p* = 0.04). In addition, the level of procalcitonin was also significantly higher in the PJP with concomitant CMV infection group than these without CMV infection group (2.2 ng/ml vs. 0.4 ng/ml, *p* = 0.004). Overall, PJP complications including pneumothorax and pneumomediastinum accounted for 12.8% (*n* = 17) and 4.5% (*n* = 6) of the 132 patients, respectively. There was no significant difference in the number of days to initiating treatment for PJP between the two groups, but 33% of the patients (*n* = 44) overall did not receive treatment. A significantly higher proportion of the PJP and CMV coinfection group received ganciclovir as anti-CMV treatment compared to these without CMV co-infection group (88.5% vs. 51.9%, *p* < 0.001).

**Table 2.** Clinical characteristics, microbiological examinations, and follow-up of patients based on CMV co-infection status.

	With CMV systemic infection (*n* = 26)	Without CMV systemic infection (*n* = 106)	*p*-value
**Disease severity**			
Severe sepsis with septic shock, n (%)	22 (84.6)	73 (68.9)	0.14
Acute respiratory distress syndrome, n (%)	18 (69.2)	76 (71.7)	0.80
APACHE II score	21.5 [11.5–23.5] (*n* = 24)	19.0 [14.5–24.0] (*n* = 104)	0.96
PaO2/FiO2 ratio	73.5 [56.3–105.0] (*n* = 26)	95.6 [74.5–146.1] (*n* = 106)	0.04
**Result of blood analysis**			
Leukocyte count (10^3^ /μL)	11.8 [7.4–42.2] (*n* = 26)	9 [5.8–16.4] (*n* = 106)	0.21
Neutrophil count (10^3^ /μL)	8.1 [5.3–27.3] (*n* = 25)	6.5 [3.8–14.6] (*n* = 100)	0.13
Presence of neutropenia (neutrophil < 1500/ μL)	0 (0)	4 (3.03)	0.58
C-reaction protein (CRP) (mg/L)	27.2 [3.1–153.9] (*n* = 26)	26.7 [4.3-99] (*n* = 104)	0.81
Albumin (g/dL)	3.1 [2.4–3.5] (*n* = 26)	3.2 [2.6–3.8] (*n* = 106)	0.13
Lactate dehydrogenase (LDH) (U/L)	403 [288.5–608.8] (*n* = 12)	384 [239-529.5] (*n* = 72)	0.47
Procalcitonin(ng/mL)	2.2 [0.5–5.3] (*n* = 23)	0.4 [0.2–1.9] (*n* = 90)	0.004
**Radiologic feature**			
Pneumothorax	4 (15.4)	13 (12.3)	0.74
Pneumomediastinum	1 (3.9)	5 (4.7)	1.00
**Pulmonary co-infection, n (%)**			
Bacterial	9 (34.6)	26 (24.5)	0.3
Fungus (*n* = 52)	4 (15.4)	9 (8.5)	0.44
virus other than CMV	4 (15.4)	10 (9.4)	0.47
**PJP treatment**			
Non treated, n(%)	7 (26.9)	37 (34.9)	–
Use TMP-SMX	19 (73.1)	69 (65.1)	0.44
Days from the index date to the TMP-SMX-used date	5 [1–9]	3 [1–5]	0.48
**Anti-CMV treatment**			
Use Ganciclovir	23 (88.5)	55 (51.9)	<0.001

Abbreviations: CMV, cytomegalovirus; PJP, *Pneumocystis jirovecii* pneumonia; APACHE, Acute Physiology and Chronic Health Evaluation; PaO2, partial pressure of arterial oxygen; FiO2, inspiratory fraction of oxygen; TMP-SMX, trimethoprim-sulfamethoxazole.

Values are listed as median [IQR] or number (%).

Categorical variables were compared by the chi-square test.

The Mann-Whitney U test was performed to compare continuous variables with abnormal distribution and given the small number of patients in subgroups.

### Outcomes

Fifty-one (38.6%) of the 132 patients died during hospitalization (Table 3), and the mortality rate was significantly higher in PJP with concomitant CMV infection group (65.4% vs. 32.1%, *p* = 0.002). In addition, 115 patients (87.1%) had ventilator-acquired pneumonia, but the difference was not significance between the two groups. Similarly, there were no significant differences in renal replacement therapy or extracorporeal membrane oxygenation between the two groups. Only 26.5% of the patients (*n* = 35) were weaned from the ventilator, and the difference was not significant between the two groups.

**Table 3.** Outcomes of the with and without CMV co-infection groups.

	With CMV systemic infection (*n* = 26)	Without CMV systemic infection (*n* = 106)	*p*-value
Ventilator acquired pneumonia	14 (53.9)	39 (36.8)	0.11
renal replacement therapy	8 (30.77)	17 (16.04)	0.09
ECMO use in ICU	1 (3.85)	3 (2.83)	1.00
Morbidity, day			
MV days	25.0 [10.0–33.0]	16.5 [10.0–29.0]	0.39
ICU days	25.0 [11.0–37.0]	19.0 [13.0–37.0]	0.74
Hospital days	31.0 [20.0–59.0]	35.5 [23.0–57.0]	0.47
Weaning ventilator, n (%)	6 (23.08)	29 (27.36)	0.66
Mortality, n (%)	17 (65.38)	34 (32.08)	0.002

Abbreviation: CMV, cytomegalovirus; PJP, *Pneumocystis jirovecii* pneumonia; ECMO, extra-corporeal membrane oxygenation; ICU, intensive care unit; MV, mechanical ventilation.

Values are listed as median [IQR] or number (%).

Categorical variables were compared by the chi-square test.

The Mann-Whitney U test was performed to compare continuous variables with abnormal distribution and given the small number patients in subgroups.

To identify independent predictors of mortality, we performed multivariate Cox regression analyses. (Table 4), and found that CMV co-infection (33.3% vs. 11.1%, *p* = 0.002), septic shock (92.2% vs. 59.3%, *p* < 0.001) and ARDS (84.3% vs. 63.0%, *p* = 0.008) were associated with an increased risk of mortality. There were no significant differences between the two groups in ventilator-acquired pneumonia, APACHE II scores or PJP treatment. In Model 1 (Adjusted Model), which adjusted for the baseline APACHE II score, systemic CMV co-infection was not significantly associated with ICU mortality (HR = 1.098, 95% CI = 0.588–2.049, *p* = 0.77). Additionally, the APACHE II score did not show statistical significance in this model. In Model 2 (Extended Model), which further incorporated clinical variables including ARDS and PJP treatment, both CMV co-infection and the APACHE II score remained non-significant. However, the development of ARDS was identified as a strong independent risk factor for mortality (HR 4.281, 95% CI:1.178–15.565, *p* = 0.027). Conversely, the duration of PJP treatment demonstrated a significant protective effect against mortality ((HR: 0.892, 95% CI: 0.803–0.990, *p* = 0.006). Septic shock showed quasi-complete separation in multivariate analysis, while it was associated with mortality in univariate analysis (HR = 6.790, 95% CI = 2.237–20.608, *p* < 0.001).

**Table 4.** Univariate and multivariate analyses of mortality during admission to the ICU.

Parameters, *n* (%)	Non-survivors (*n* = 51)	Survivors (*n* = 81)	Univariate analysis *p*-value	Model 1^b^ Adjusted HR (95% CI)	*p*-value	Model2^c^ Extended HR‡ (95% CI)	*p*-value
CMV co-infection	17 (33.3)	9 (11.1)	0.002	1.098 (0.588–2.049)	0.77	1.055 (0.337–3.300)	0.93
**Other pulmonary co-infection**							
Bacterial infection	15 (29.4)	20 (24.7)	0.55			1.923 (0.734–5.036)	0.18
Fungus	7 (13.7)	6 (7.4)	0.24			1.901 (0.475–7.604)	0.36
Virus other than CMV	4 (7.8)	10 (12.4)	0.56			0.824 (0.169–4.012)	0.81
**Follow up complications**							
Acute respiratory distress syndrome	43 (84.3)	51 (63.0)	0.008			4.281 (1.178–15.565)	0.027
PJP treatment duration, days	10 [3–15]	10 [5–15]	0.62			0.892 (0.822–0.968)	0.006
Ventilator Acquired pneumonia	24 (47.1)	29 (35.8)	0.20				
**Disease severity**							
APACHE score	20.5 [15–24]	18 [13.5–23]	0.19	1.048 (0.989–1.109)	0.11	1.056 (0.982–1.136)	0.14
Septic shock	47 (92.2)	48 (59.3)	<0.0001	^ad^			

Abbreviation: HR, Hazard ratio; CMV, cytomegalovirus; PJP, Pneumocystis jirovecii pneumonia; APACHE, Acute Physiology and Chronic Health Evaluation;

Values are listed as median [IQR] or number (%).

^a^Found Quasi-complete separation

^b^Model 1: Adjusted for baseline confounders (disease severity at admission).

^c^Model 2: Extended model additionally including variables that may lie on the causal pathway (e.g., ARDS, septic shock, and treatment-related variables).

^d^Septic shock showed quasi-complete separation and was not estimable in the extended model.

## Discussion

This study adds to the growing body of evidence regarding PJP and CMV co-infection in non-HIV critically ill patients. Our results indicate that concomitant PJP and CMV systemic infection is a serious issue in these patients, and that it is associated with substantial mortality. In addition, we found that concomitant PJP and CMV infection was associated with more severe disease including a lower PF ratio, but that there was no significant association with mortality in multivariate analysis. However, mortality was independently associated with septic shock, ARDS and PJP treatment.

Our study demonstrated clinical characteristics largely consistent with prior epidemiological reports [2,3,5,23]. PJP in our cohort predominantly occurred in immunocompromised patients, particularly those with malignancies, with 46% having solid tumors and 10% hematologic malignancies. Corticosteroid exposure, a well-established risk factor for PJP, was also common in non-HIV patients [24], with a prevalence of 84–98% of steroid use in patient of PJP with respiratory failure [6,25–27]. 75% of our enrolled patients having received steroids while pneumothorax occurred at a rate consistent with previous reports (5–20%) [26,28]. In addition, disease severity was high in our cohort, with 70.3% presenting with septic shock and 71% progressing to ARDS, compatible with previous studies [4–8]. Mortality risk was increased in PJP patients accompanied by elevated severity scores (e.g. APACHE II, SOFA) and impaired oxygenation (low PF ratio) [16,24,29]. The ICU mortality rate was 45.7% in our cohort, which, despite the high severity, remained slightly lower than previously reported rates (52–76%) in comparable ICU populations [2,16,29–34].

CMV co-infection with PJP has been discussed for a long time, initially focusing on HIV-infected patients [29,35]. There is growing interest in non-HIV patients not only due to the increasing incidence of PJP, but also because of its theoretical background; for example, more severe lung disease was observed in a mouse model of CMV and pneumocystis co-infection [14]. Some studies have reported a prevalence of CMV infection in the ICU of about 18-35%, and that it was associated with prolonged ICU stay and increased mortality [36–38]. Previous studies have reported a prevalence of CMV pulmonary co-infection in patients with PJP ranging from 27% to 61% [12,13,29,39]. In our study, the prevalence of CMV co-infection with PJP was 19.7%. Although this is lower compared to other studies, the discrepancy may be related to varying definitions of CMV infection across studies.

Regarding the manifestations of PJP and CMV co-infection, we found that the PJP with concomitant CMV infection group had a high prevalence of septic shock and statistically lower PF ratio and elevated procalcitonin levels, indicating that the PJP with concomitant CMV infection group had more severe pneumonia than these without CMV infection group. Consistent with our findings, previous studies have also reported higher rates of mortality, with significant elevations in procalcitonin and CRP levels and a lower PF ratio, potentially reflecting higher disease severity [12,39,40]. Existing literature regarding the impact of CMV co-infection on mortality in patients with PJP has yielded conflicting results. While some studies have reported a significant association between CMV and poor outcomes including an increased risk of ICU admission, need for invasive ventilation, ARDS, longer ICU stay, and higher mortality rate [12,13,24], others, particularly those focusing on critically ill populations, have found no such correlation [15–17,41,42]. Despite our limitation to detect BALF CMV DNA, we checked plasma CMV DNA when the patient admitted to ICU and set a higher cut-off level as possible systemic infection [10], trying to distinguish from CMV reactivation [43]. Additionally, CMV infection and disease are predominantly defined by consensus criteria established in transplant patient populations [44], there still need more studies to verify the role of CMV infection or reactivation in critical-ill population patient because of increased risk of mortality and ICU stay [37,45].

From a methodological standpoint, these discrepancies may arise not only from variations in diagnostic definitions but also from a lack of clarity in distinguishing between confounders and mediators within the causal framework. According to the principles of causal inference, adjusting for clinical complications that lie downstream on the causal pathway—such as acute respiratory distress syndrome (ARDS) or septic shock—can introduce overadjustment bias, thereby masking the total effect of the primary exposure [46]. In the present study, however, our hierarchical analysis demonstrated that even in the adjusted model (Model 1), which controlled for baseline physiological status *via* the APACHE II score, systemic CMV co-infection remained statistically non-significant. This finding provides possible explanation to the ongoing academic debate: in non-HIV, critically ill patients with PJP, systemic CMV infection likely serves as a surrogate marker for profound immunosuppression and overall disease severity, rather than acting as an independent driver of mortality.

The clinical implications of this finding are further reflected in our analysis of treatment and complications. In the treatment of PJP, delayed diagnosis and treatment have been associated with poor outcomes [26,47,48]. Although about 33% (*n* = 44) of our patients did not receive anti-*P. jirovecii* treatment, our multivariate analysis demonstrated that the duration of PJP treatment is still a significant independent protective factor against mortality. Regarding CMV co-infection, although ganciclovir was administered to a significantly higher proportion of patients in the co-infected group, its use did not translate into a survival benefit, and CMV remained non-significant in the extended model (*p* = 0.93). Kim et al. reported that PJP and CMV co-infected patient with lower CMV T cell-mediated immunity had higher mortality, even in those who used ganciclovir [41], which could also support with our inference. Instead, the prognosis was influenced by the development of ARDS and Septic shock in our study after multivariate analysis, which is compatible with previous discussion that CMV reactivation or infection in PJP patient may be an indicator of stronger immunosuppression status and illness severity [12,14,16,41]. However, there are still lack of data in this patient group because of different study protocols and patient groups enrolled. Additionally, there is currently no treatment guidelines or consensus regarding this specific situation focusing on critical-ill or mechanical ventilated patient, so the use of ganciclovir in our retrospective study was applied heterogeneously, which may have still biased our mortality analysis. Therefore, to improve the management of PJP patient with CMV co-infection, further studies are needed to determine the impact of CMV treatment and the associated CMV titre cut-off values in either serum or respiratory samples in different patient group.

Our study has several limitations. First, as this was a retrospective and observational study, we could not thoroughly assess some data including the duration and dosage of steroids or other immunosuppressants and also medications for CMV prophylaxis when PJP was diagnosed. Second, because of the limitations in diagnosing PJP (including long testing times, no beta-D-glucan (BDG) test, image evaluation), some of our patients did not receive timely treatment or did not receive prompt second-line anti-pneumocystis treatment, which may have influenced our prognosis analysis. Third, our inclusion criteria required BAL for definitive diagnosis. This inherently excludes patients with severe hypoxemia who were deemed too unstable for the procedure, potentially selecting for a specific phenotype of ICU patients. Fourth, we could not measure the CMV viral load in BAL fluid owing to our limitation of diagnostic capabilities, which may have biased our assessment of CMV infection status. We could not measure the CMV viral load in BAL fluid owing to our limitation of diagnostic capabilities, which may have biased our assessment of CMV infection status. Furthermore, regarding the definition of CMV infection, we used a plasma CMV DNA cut-off value > 2000 IU/mL to define active systemic infection, which may have misestimated the severity of the patients’ condition and affected the efficacy of our treatment interventions. This may produce the selection bias and collider bias. In addition, though CMV viral load was analyzed when patient admitted to ICU, there was no baseline CMV serology of the patients before the diagnosis of PJP, and thus we could not evaluate whether the CMV status was due to reactivation or primary infection, which may have interfered with our outcome analysis and interpretation. Finally, as previously stated, no specific antiviral guidelines exist regarding this situation, and our antiviral treatment may have been applied heterogeneously.

## Conclusion

In conclusion, about one-fifth of PJP patients receiving invasive mechanical ventilation having concomitant CMV infection. Our multivariate analysis demonstrated that systemic CMV co-infection was not an independent predictor of mortality. Patient prognosis was primarily driven by the development of septic shock, ARDS and PJP treatment. These findings suggest that CMV viremia in this population may likely act as a surrogate marker for disease severity rather than a direct pathogenic driver. Therefore, clinical management should prioritize the early initiation and keep management duration of adequate anti-pneumocystis therapy and the implementation of lung-protective strategies for ARDS. [Future large-scale studies and application are still needed to evaluate the therapeutic benefit of anti-CMV treatment in patients with PJP and CMV co-infection.

## Data Availability

The data will not be shared according to the regulations of Chang Gung Memorial Hospital IRB for patient confidentiality.
